# Delayed duodenal stump fistula after laparoscopic distal gastrectomy with Billroth-II reconstruction for early gastric cancer: A case report

**DOI:** 10.1097/MD.0000000000029732

**Published:** 2022-07-08

**Authors:** Yeo Jin Kim, Dae Hoon Kim, Hanlim Choi, Dong Hee Ryu, Hyo Yung Yun

**Affiliations:** a Department of Surgery, Chungbuk National University Hospital, Chungcheongbuk-do, Republic of Korea; b Department of Surgery, Chungbuk National University College of Medicine, Chungcheongbuk-do, Republic of Korea.

**Keywords:** distal gastrectomy, duodenal stump fistula, laparoscopy, metallic surgical clip

## Abstract

Duodenal stump fistula (DSF) is one of the most serious complications of gastrectomy. The mean time to diagnosis of DSF is approximately 9 days after operation. Our report describes an extremely rare case of delayed DSF 144 days after a laparoscopic distal gastrectomy.

A 58-year-old man with drug-induced liver cirrhosis (Child-Pugh class A) underwent laparoscopic distal gastrectomy with Billroth-II reconstruction for early gastric cancer. On postoperative day 1, he underwent reoperation because of intra-abdominal bleeding. Ongoing bleeding was observed in the stapler line of the duodenal stump and was controlled using metallic surgical clips. The patient was discharged on postoperative day 14, without complications. After 144 days following the first operation, he visited the emergency room with right flank pain and high fever. Computed tomography revealed free air and abscess near the duodenal stump site.

Emergency laparotomy, abscess unlooping, and drain insertion were performed. After surgery, bile was drained by intra-abdominal drainage, and fistulography showed a duodenal fistula.

The patient was discharged 55 days after his third surgery.

This is an extremely rare case of DSF, which may be caused by the metallic surgical clips used for hemostasis of the duodenal stump stapler line. We believe that the use of metallic surgical clips for hemostasis of the duodenal stump after Billroth-II reconstruction should be avoided.

## 1. Introduction

Recently, the survival rate of gastric cancer patients has improved because of the increase in the diagnosis of early gastric cancer, the decrease in operative mortality, and the improvement of adjuvant chemotherapy.^[1]^ However, gastric cancer is still the most common cancer and is the fourth most common cause of cancer death in South Korea.^[2]^ Advances have been made in gastric cancer surgery to improve the outcome, but the treatment of gastric cancer is still a high-risk surgery.^[3]^ Duodenal stump fistula (DSF) is a rare but dreadful complication after distal or total gastrectomy. The incidence of duodenal fistula is 1.6%–2.7%, and the mortality rate is 16%–40%.^[4–6]^ Inflammation, inadequate duodenal stump closure, R1 or R2 resection of duodenal stump, incorrect drain position, devascularization, postoperative duodenal distension, and local hematoma are known risk factors for DSF.^[4,6]^ The mean interval of DSF after gastrectomy is 6.6–9 days.^[5,7]^ Long-term interval of DSF is rare. One patient was reported to have delayed DSF after total gastrectomy for gastric cancer. The interval time of DSF was 45 days after the operation, and it was related to afferent loop syndrome.^[8]^ To date, there have been no reported cases of delayed DSF that are not related to distal intestinal obstruction. Recently, we experienced delayed DSF without afferent loop syndrome or distal intestinal obstruction 144 days after the first operation, which was caused by metallic surgical clips used for hemostasis in the duodenal stump.

## 2. Patient Consent Statement

This study was approved by the Institutional Review Board of the Chungbuk National University Hospital, Republic of Korea. The patient signed an informed consent form for the publication of this case report and any accompanying images.

## 3. Case Presentation

**Figure 1.**
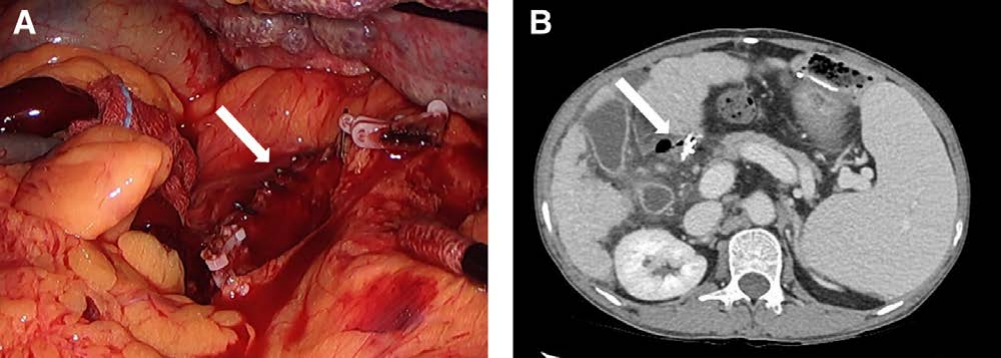
(A) Metallic surgical clips were applied to the duodenal stump for hemostasis (white arrow). (B) Free air is seen around the duodenal stump, and a metallic surgical clip is seen in the duodenal stump stapler line (white arrow).

A 58-year-old man with drug-induced liver cirrhosis (Child-Pugh class A) underwent laparoscopic distal gastrectomy with Billroth-II reconstruction for early gastric cancer. During gastrectomy, bleeding in the duodenal stump was controlled using metallic surgical clips (Fig. 1A). On postoperative day (POD) 1, he underwent reoperation because of intra-abdominal bleeding. Bleeding was observed in the stapler line of the duodenal stump, pancreatic head, and mesocolon. Bleeding of the duodenal stump was controlled by applying more metallic surgical clips to the stapler line. The patient was discharged on POD 14 without complications. He visited the outpatient clinic after 3 months. There were no abnormal laboratory findings or physical examination results.

He was transferred to our emergency room 144 days after the first operation because of a high fever. At the emergency room, he had a high fever of 39.0°C but had no other physical examination. In addition, there were no abnormal laboratory findings except elevated high sensitivity C-reactive protein (hsCRP) of 3.34 mg/dL. In the emergency room, he was discharged after receiving medication for upper respiratory tract infection.

However, he revisited the emergency room the next day with right flank pain and a high fever of 39.6°C which occurred 12 hours before the return to the emergency room. Additionally, the patient complained of general weakness. There were no abnormal laboratory findings except an elevated hsCRP of 4.80 mg/dL. He underwent abdominal and pelvic computed tomography (CT), which showed free air and an abscess around the duodenal stump, which was enhanced with metallic surgical clips that had been used during previous bleeding control surgery (Fig. 1B). Emergency laparotomy was performed. The abdominal cavity was thoroughly explored, and severe adhesions were observed in the mesocolon, omentum, and liver. Moreover, severe inflammatory changes and abscess formations were observed between the gallbladder and the duodenal stump. We could not clearly identify the duodenal stump because of bleeding, severe adhesions, and inflammatory changes. We performed partial transverse colectomy, cholecystectomy, and abscess unlooping with the insertion of a Jackson-Pratt drain.

**Figure 2.**
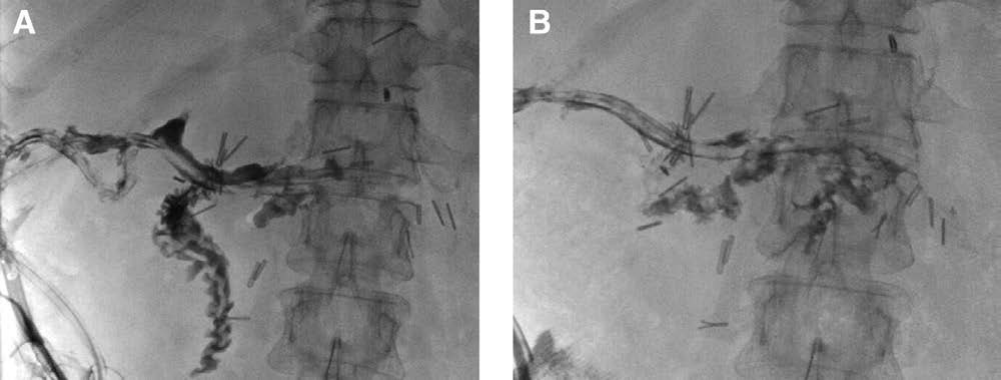
(A) Fistulography on POD 14. Intraluminal contrast media flow is identified on fistulography via Jackson-Pratt drainage catheter suggesting DSF. (B) Follow-up fistulography on POD 29. There is intraluminal contrast media flow into the duodenal stump but with partial visualization and no passage to distal duodenum suggesting duodenal stump fistula improvement. DSF = duodenal stump fistula, POD = postoperative day.

On POD 2, bile was drained through Jackson-Pratt drainage. On POD 14, fistulography was performed, and DSF was identified (Fig. 2A). After conservative management, follow-up fistulography performed on POD 29 showed an improvement in the DSF (Fig. 2B). The patient was discharged POD 55, with no other complications.

## 4. Discussion

Recently, surgical outcomes after gastrectomy for gastric cancer have improved, contributing to better outcomes in patients with gastric cancer.^[1]^ These patients are mostly treated using the laparoscopic approach, which plays a role in the improvement of short-term postoperative outcomes.^[3]^ In a large-scale multicenter randomized controlled trial for laparoscopic distal gastrectomy in Korea (KLASS-01),^[9]^ the incidence of postoperative morbidity was significantly lower in laparoscopic distal gastrectomy than in open distal gastrectomy. Although laparoscopic distal gastrectomy has fewer complications than open conventional distal gastrectomy, there were still 13.7% of the patients who experienced postoperative morbidity, and 1.2% underwent reoperation due to postoperative complications.

DSF is defined as the presence of duodenal fluid through an intra-abdominal drain or its leakage through the abdominal wall, and it can be detected by abdominal CT or fistulography.^[5,10]^ Although the presence of DSF alone is no longer considered to cause death in gastric cancer patients after gastric surgery, DSF can be life-threatening when combined with other complications, especially sepsis.^[4]^ Previously, the overall incidence of DSF was reported to be 1.8%–3%, and the DSF-related mortality rate was reported to be 7%–67%.^[11–13]^ However, recent studies reported the incidence of duodenal fistula to be 1.6%–2.7%, and the mortality rate to be 16%–40%.^[4–6]^ This decrease in the incidence and mortality of DSF may be due to technical improvements in gastric cancer treatment. Inflammation, inadequate duodenal stump closure, R1 or R2 resection of the duodenal stump, incorrect drain position, devascularization, postoperative duodenal distension, and local hematoma are known risk factors for DSF.^[4,6]^ In addition, comorbidities and gastric outlet obstruction before surgery can also be significant risk factors for DSF.^[7]^

Medical therapy is the treatment of choice for this condition. However, other treatments are available, including surgical, percutaneous, and endoscopic approaches. Medical therapy involves parenteral nutrition and administration of drugs such as antibiotics, somatostatin, or octreotide. Often, medical therapy is associated with the percutaneous approach, which involves treatments such as percutaneous drainage of the abscess, percutaneous duodenostomy, and percutaneous transhepatic biliary drainage. The surgical approach involves pancreatoduodenectomy and the use of a rectus muscle flap.^[4,6]^ The endoscopic approach involves techniques such as endoscopic stitching, stapling, biological glue, diversion with stent, and endoscopic vacuum-assisted closure.^[14]^

The mean interval of DSF after gastrectomy is 6.6–9 days.^[5,7]^ A case report which reported a delayed DSF after 45 days of initial surgery had explained it was caused by increased backpressure on the afferent limp and duodenal stump due to obstruction of the distal jejunum.^[7]^ The complications of metallic surgical clips, such as allergic reactions,^[15]^ foreign body reactions,^[16]^ and migrations,^[17,18]^ are known. There have been no reports to date that metallic surgical clips are associated with fistulas of the hollow viscus. In the present case, we used an excessive amount of metallic surgical clips for hemostasis of the duodenal stump. After 144 days of laparoscopic distal gastrectomy, free air around the metallic surgical clips was observed on CT, suggesting DSF which was confirmed in the fistulogram obtained after surgery. It is presumed that the excessive use of metallic surgical clips for hemostasis of the duodenal stump may have caused DSF. The duodenal stump is surrounded by other tissues, such as the gall bladder, mesocolon, and liver. Therefore, metallic surgical clips on the duodenal stump may have caused pressure on the stapler line. For this reason, DSF caused by metallic surgical clips might have progressed very slowly, eventually showing its presence 144 days after the first operation.

## 5. Conclusion

Even if surgery was performed a long time ago, if clinical or radiologic findings suggest DSF, it should not be overlooked. Moreover, surgeons should be cautious when using metallic surgical clips for hemostasis of duodenal stump bleeding because metallic surgical clips can cause delayed DSF.

## Acknowledgments

We would like to thank Editage (www.editage.com) for English language editing.

## Author contributions

Conceptualization: Dae Hoon Kim

Data curation: Yeo Jin Kim, Dae Hoon Kim

Investigation: Dae Hoon Kim

Supervision: Hanlim Choi, Dong Hee Ryu

Writing—original draft: Yeo Jin Kim

Writing—review and editing: Ryu Dong Hee, Hanlim Choi
